# The immune response to sub-clinical mastitis is impaired in HIV-infected women

**DOI:** 10.1186/s12967-018-1667-4

**Published:** 2018-10-25

**Authors:** Roxane Schaub, Stéphanie Badiou, Johannes Viljoen, Pierre Dujols, Karine Bolloré, Philippe Van de Perre, Marie-Louise Newell, Ruth Bland, Nicolas Nagot, Edouard Tuaillon

**Affiliations:** 1 Pathogenesis and Control of Chronic Infections, INSERM, EFS, Université Montpellier 1, Montpellier, France; 2 Département d’Information Médicale, CHU de Montpellier, Montpellier, France; 3 Département de Biochimie, CHU de Montpellier, Montpellier, France; 4 Africa Centre for Health and Population studies, University of KwaZulu-Natal, Durban, South Africa; 5 Department of Medical Virology, University of Pretoria and NHLS, Pretoria, South Africa; 6 Département de Bactériologie-Virologie, CHU de Montpellier, Montpellier, France; 7 Institute for Developmental Science, Human Development and Health, Faculty of Medicine, University of Southampton, Southampton, UK; 8 School of Public Health, Faculty of Health Sciences, University of Witwatersrand, Johannesburg, South Africa; 9 Royal Hospital for Children, Institute of Health and Wellbeing, University of Glasgow, Glasgow, UK; 10 Present Address: CIC AG/Inserm 1424, Centre Hospitalier de Cayenne, Av. des flamboyants, BP 6006, 97 306 Cayenne CEDEX, French Guiana France

**Keywords:** HIV, Breast milk, Subclinical mastitis, Cytokines

## Abstract

**Background:**

Subclinical mastitis (SCM) is relatively common in lactating women and may be associated with HIV shedding in breast milk. The potential association between HIV infection and breast milk immunologic factors and immune response to SCM needs to be addressed.

**Methods:**

In this cross-sectional study, SCM (Na/K ratio > 1) was tested in 165 mature breast milk samples collected from 40 HIV-infected women who didn’t transmit HIV to their child by breastfeeding and 43 HIV-uninfected women enrolled in an interventional cohort in South-Africa (Vertical Transmission Study). The level of 33 immune markers related to Th1/Th2 related response, inflammation and bacterial exposure were compared in ART-naive HIV-infected versus HIV-uninfected women. The associations between HIV infection and SCM on the concentration of immune factors were tested separately by Wilcoxon rank-sum test and corrected for false discovery rate. To control for potential confounder effects and take into account the clustering of breast milk samples from a single woman, multivariate mixed linear models adjusted on child age at the time of sampling were performed for each immune factor.

**Results:**

Subclinical mastitis was detected in 15 (37.5%) HIV-infected women and 10 (23.3%) HIV-uninfected women. In the absence of SCM, the breast milk levels of IP-10 and MIG were higher and IL1-RA lower in HIV-infected women than in HIV-uninfected women (respectively p < 0.001, p = 0.001, p = 0.045). In HIV-uninfected women, SCM was characterized by a robust immune response with higher concentrations of a broad panel of Th1 and inflammatory related immune markers than in samples without SCM. By contrast, in HIV-infected women a limited number of immune markers were increased and lower increases were observed in samples with SCM than without SCM.

**Conclusion:**

HIV infection in ART-naïve women was associated with elevated breast milk levels of IP-10 and MIG, which areTh1-related cytokines induced by IFN-γ. During SCM, a lower and narrower immune response was observed in HIV-infected than HIV-uninfected women, suggesting that HIV infection affects the capacity of the mammary gland to respond to SCM.

**Electronic supplementary material:**

The online version of this article (10.1186/s12967-018-1667-4) contains supplementary material, which is available to authorized users.

## Background

Mother-to-child HIV transmission (MTCT), can occur during pregnancy, delivery or breastfeeding and remains a public health concern especially in sub-Saharan Africa. Despite an overall 48% decrease of newly infected children between 2009 and 2014 due to the implementation of prophylaxis interventions and antiretroviral therapy (ART), there were still 160,000 newly infected children (< 15 years old) in 2016 [1]. Postnatal HIV transmission through breastfeeding in the presence of ART contributes to a significant part of this residual transmission with a pooled risk estimate of 2.93% at 12 months [2]. World Health Organization (WHO) guidelines recommend lifelong ART for mothers living with HIV [3]; exclusive breastfeeding for the first 6 months of life; appropriate complementary foods thereafter and to continue breastfeeding for at least 12 months [4].

Breast milk, the main and optimal nutrient source of the infant, contains numerous active immune factors [5, 6]. These immune factors protect the infant from infections [7], participate in the maturation of the infant’s immune system [6], down-regulate gut inflammation and promote gut adaptation after birth when the newborn is confronted with the antigens of colonizing commensal bacteria [8]. Breast milk soluble immune factors consist of a wide array of bioactive agents like cytokines, chemokines, growth factors and acute phase proteins [6, 9]. These immune factors are involved in multiple immune functions including Th1 response, antimicrobial, anti-inflammatory, pro-inflammatory and immunomodulatory properties [5, 6, 9, 10] and influence the immune response by attracting, activating or down-regulating different cell effectors [9, 10]. During lactation, inflammatory processes of the mammary gland like mastitis [11–15] and subclinical mastitis (SCM) [16–19] induce considerable changes in the breast milk immunefactors. In asymptomatic lactating women, SCM is generally defined by an increased breast milk sodium/potassium ratio (Na/K > 1) [17, 18, 20, 21]. Whereas clinical mastitis occurs in less than 10% of lactating mothers [22–24], SCM (Na/K > 1) is more frequent, especially at start of breastfeeding in the first few days after delivery and again at the time of weaning, with a prevalence ranging from 9 to 45% in mature milk in HIV-uninfected mothers [17, 18]. Studies have suggested that SCM may be associated with HIV shedding in breast milk and HIV mother-to-child transmission [25–34].

Human immunodeficiency virus is known to induce immune activation in blood [35], gut [36] and female genital tract [37, 38] but its impact on milk immune factors remains only partially defined. Few studies have compared breast milk components in ART-naïve HIV-1-infected versus HIV-uninfected women, and a limited number of parameters were assessed [39–41]. We hypothesized that HIV infection induces modifications of the composition of immune factors in breast milk, and impairs immune response to SCM in breast milk.

In a first step, our study assessed the influence of HIV infection (without ART exposure) on the pattern of breast milk immune factors. We further sought to explore the association between HIV infection and immune response to SCM.

## Materials and methods

### Study population and clinical features

This study was part of the ANRS 1271 project exploring factors associated with HIV-1 transmission from mother to child through breastfeeding. The study was nested in a South-African non-randomized prospective intervention cohort, the Vertical Transmission Study (VTS), examining the effect of feeding practices on infant HIV infection and survival rates, in a community where HIV prevalence in pregnant women was 23.7% [42]. A total of 2722 HIV-infected and uninfected pregnant women attending antenatal clinics in KwaZulu-Natal were enrolled after providing informed consent between October 2001 and April 2005 [43]. Participants were naive to antiretroviral therapy except for single-dose nevirapine provided to all HIV-infected women and their newborns during delivery as per national guidelines at the time. The intervention included a personalized antenatal counseling session on infant feeding choices, and a postnatal home-based breastfeeding counseling intervention for women who chose to breastfeed. Formula feeding women were supported at their clinic visits by study nurses. Daily infant feeding practices and maternal breast health problems or breastfeeding difficulties were recorded. Maternal health and infant growth and morbidity were regularly recorded in monthly scheduled clinic visits until 9 months and every 3 months afterwards until 2 years of age. Mother’s blood samples were taken before delivery and at 6 months post-delivery to measure HIV viral load and CD4 count and child’s HIV status was checked at each study clinic visit on dried blood spots. Breast milk samples were taken separately from right and left breast at each clinic visit if the mother was still breastfeeding [44]. The VTS study and breast milk analyses were approved by the Biomedical Research Ethics Committee of the University of KwaZulu-Natal and women gave informed consent.

Forty HIV-infected women who did not transmit HIV to their infant during follow-up and 43 HIV-uninfected women were enrolled for this study. Subjects were selected based on availability of mature breast milk samples (breast milk collected > 15 days after delivery) and in a random draw from the VTS cohort. Breast milk samples from HIV-infected women who transmitted HIV to their child were excluded in our study to focus on the impact of HIV infection on breast milk immune factors and on response to SCM. A total of 156 breast milk samples were tested. Paired left and right breast samples were available for 73 (88.0%) women.

SCM was defined by a Na/K ratio in breast milk > 1, as described elsewhere [17, 18, 45, 46]. Maternal breast health problems (such as mastitis, engorgement, cracked/bleeding nipple, blocked duct, breast thrush, breast/nipple oozing pus and abscess [24]) were considered if they occurred during the week before the sample collection date because mastitis markers are normalized within 1 week after symptom resolution [13]. Breastfeeding practices were defined as in the VTS cohort [43], but were only included on the 15 days before the sample collection date, in order to detect a recent breastfeeding practice change that may be indicative of a breast health problem [47].

We explored four groups of breast milk samples: samples without SCM from HIV-uninfected women, samples with SCM from HIV-uninfected women, samples without SCM from HIV-infected women and samples with SCM from HIV-infected women.

### Immune factors and biochemical assays in breast milk

Whole breast milk samples were stored at − 80 °C until processing at the Montpellier University Teaching Hospital, France. All parameters were measured in lactoserum after centrifugation at 1200*g* for 15 min. Lactoserum sodium and potassium concentrations were measured with ion selective electrode (AU640 analyzer, Beckman Coulter, Fullerton, CA). β2microglobuline (B2M)and C-reactive protein (CRP) were determined by immunoturbidimetric methods (AU640, Beckman Coulter, Fullerton, CA). Lactoserum erythropoietin (EPO) (IMMULITE2000 EPO assay, Diagnostic Products Corporation, Los Angeles, CA), α-defensin (Hycult Biotech, Uden, The Netherlands), lactoferrin (Calbiochem, Dramstadt, Germany), secretory leukocyte peptidase inhibitor (SLPI) (R&D Systems, Minneapolis, MN), lipopolysaccharide-binding protein (LBP) (Hycult Biotech), soluble CD14 (sCD14) (Hycult Biotech) and S100A9 protein (PS100A9) (CycLex, Nagano, Japan) were quantified by commercial enzyme immunoassays as recommended by the manufacturer. Interleukins (IL) 1β, 2, 4, 5, 6, 7, 8 (IL-8 or CXCL8), 10, 13, 15 and 17, interleukin 12p40/70, receptor antagonist of interleukin 1β (IL-1RA), interleukin 2 receptor (IL-2R), granulocyte and macrophage growth factor (GM-CSF), tumor necrosis factor- α (TNF-α), γ interferon (IFN-γ), α interferon (IFN-α), macrophage inflammatory protein 1α and 1β (MIP-1α and MIP-1β), inflammatory protein 10 (IP-10 or CXCL10), monokine induced by gamma interferon (MIG or CXCL9), eotaxin, regulated upon activation normal T-cell expressed and secreted (RANTES or CCL5), and monocyte chemotactic protein-1 (MCP-1 or CCL2) were quantified using a multiplex microbeads assay (Invitrogen Human Cytokine 25-Plex Panel, Marne-La-Vallée, France) and a Luminex 100 apparatus (Luminex, Oosterhout, The Netherlands) following the manufacturer’s instructions.

Concentrations below the lower limit of quantification were assigned half the value of the lower limit, as described elsewhere [39, 48]. Conversely, concentrations above the upper limit of quantification were assigned the value of the upper limit [17]. Immunologic factors for which 50% or more of the samples in a group were below the lower limit of quantification of the test weren’t quantitatively analyzed.

### Statistical analyses

In the first step of the study, breast milk samples were tested for SCM based on Na/K ratio. Then, the association between ART-naïve HIV infection and breast milk environment in the absence of SCM were analyzed by comparing concentrations of immune markers in samples from ART-naïve HIV-infected versus HIV-uninfected women. Next, changes induced by SCM were explored by comparing levels of immune factors in SCM samples to breast milk samples without SCM in ART-naïve HIV-1 infected and uninfected women.

Demographic and clinical characteristics of women, immunologic factors detection rates and concentrations were compared between HIV positive and negative women using the Chi-square or Fisher exact test for qualitative variables and the Student or Wilcoxon rank-sum test for quantitative variables according to the variable’s distribution. The associations between HIV infection and SCM on immune factor’s concentration were tested separately by Wilcoxon rank-sum test. Spearman’s non parametric test was used to assess correlation between immunologic factors by group. When immunologic factors were compared in sets of bivariate analyses, p-values were corrected for false discovery rate (FDR; *p *< 0.05) to correct for multiple testing [49].

To control for potential confounder effects, multivariate mixed linear models were performed for each immunologic factor. Models were done with the SAS PROC MIXED procedure, adjusted on child age at the time of sampling [48, 50–52] and taking into account the clustering of left and right breast milk samples of a woman by introducing a random effect. All statistical analyses were conducted using SAS statistical software version 9.2 (SAS Institute, Cary, NC).

## Results

### Characteristics of women and breast milk samples

Demographic, obstetrical and clinical characteristics did not differ significantly between the 40 HIV-infected women and the 43 HIV-uninfected women (Table 1). None of the women reported any breast health problem in the week preceding the sample collection. A sub-clinical mastitis (Na/K ratio > 1) in at least one breast milk sample was detected in 15/40 (37.5%) HIV-infected women and 10/43 (23.3%) HIV-uninfected women. SCM was bilateral (in samples of both left and right breast at the same time) in 4/15 (26.7%) HIV-infected women and in 5/10 (50.0%) HIV-uninfected women.

**Table 1:** Maternal demographic, obstetrical and clinical characteristics

Characteristics	HIV+ women (n = 40) Median [range] or N (%)	HIV− women (n = 43) Median [range] or N (%)
Maternal age at delivery (years)	26 [17–39]	23 [17–46]
Previous pregnancies (at least one)	30 (75.0%)	25 (58.1%)
Mode of delivery		
Vaginal	36 (90.0%)	38 (88.4%)
Caesarean	4 (10.0%)	5 (11.6%)
Infant age at breast milk sampling (days)	176 [38–494]	162 [31–493]
Breastfeeding type until sampling		
EBF^a^	20 (50.0%)	17 (39.5%)
MBF^b^	12 (30.0%)	18 (41.9%)
Unknown	8 (20.0%)	8 (18.6%)
Breastfeeding type during the past 15 days before sampling		
EBF^a^	24 (60.0%)	25 (58.1%)
MBF^b^	16 (40.0%)	18 (41.9%)
Sub-clinical mastitis in at least 1 sample (Na/K > 1)	15 (37.5%)	10 (23.3%)
Postnatal CD4-cell count (per μL)^c^	595 [78–2473]	–
Postnatal plasma HIV viral load (copies/mL)^c^	5300 [25–110,000]	–

^a^EBF: exclusive breastfeeding (breast milk only)

^b^MBF: mixed breastfeeding (MBF is defined as breast milk plus 1 day of solid food and/or breast milk plus 3 days of fluids other than breastmilk)

^c^Approximately 6 months after delivery

Subclinical mastitis was detected in 34/156 breast milk samples (21.8%): 19/74 (25.7%) samples from HIV-infected women and 15/82 (18.3%) from HIV-uninfected women. In the breast milk of HIV-infected women, HIV RNA was detected in 6/69 samples (8.7%; ranging from 615 to 22,428 copies/mL).

The number of samples analyzed by group included 67 samples without SCM from HIV-uninfected women, 15 samples with SCM from HIV-uninfected women, 55 samples without SCM from HIV-infected women and 19 samples with SCM from HIV-infected women.

Seven breast milk immune factors out of 34 were detected over the lower limit of quantification in all samples (IL-8, IP-10, EPO, MCP-1, LBP, sCD14 and B2M) and 13 were quantified in at least half of the samples (IL-2 receptor, IL-12p40/70, IL-15, MIG, IL-7, Lactoferrin, MIP-1α, MIP-1β, SLPI, RANTES, CRP, PS100A9, IL-1RA) (see Additional file 1: Table S1 for immune factors detection rates by group). Fourteen immune factors were not further considered because their concentrations were below the lower limit of quantification in the majority of the samples.

### Association between HIV infection and breast milk immune factors in the absence of SCM

**Fig. 1:**
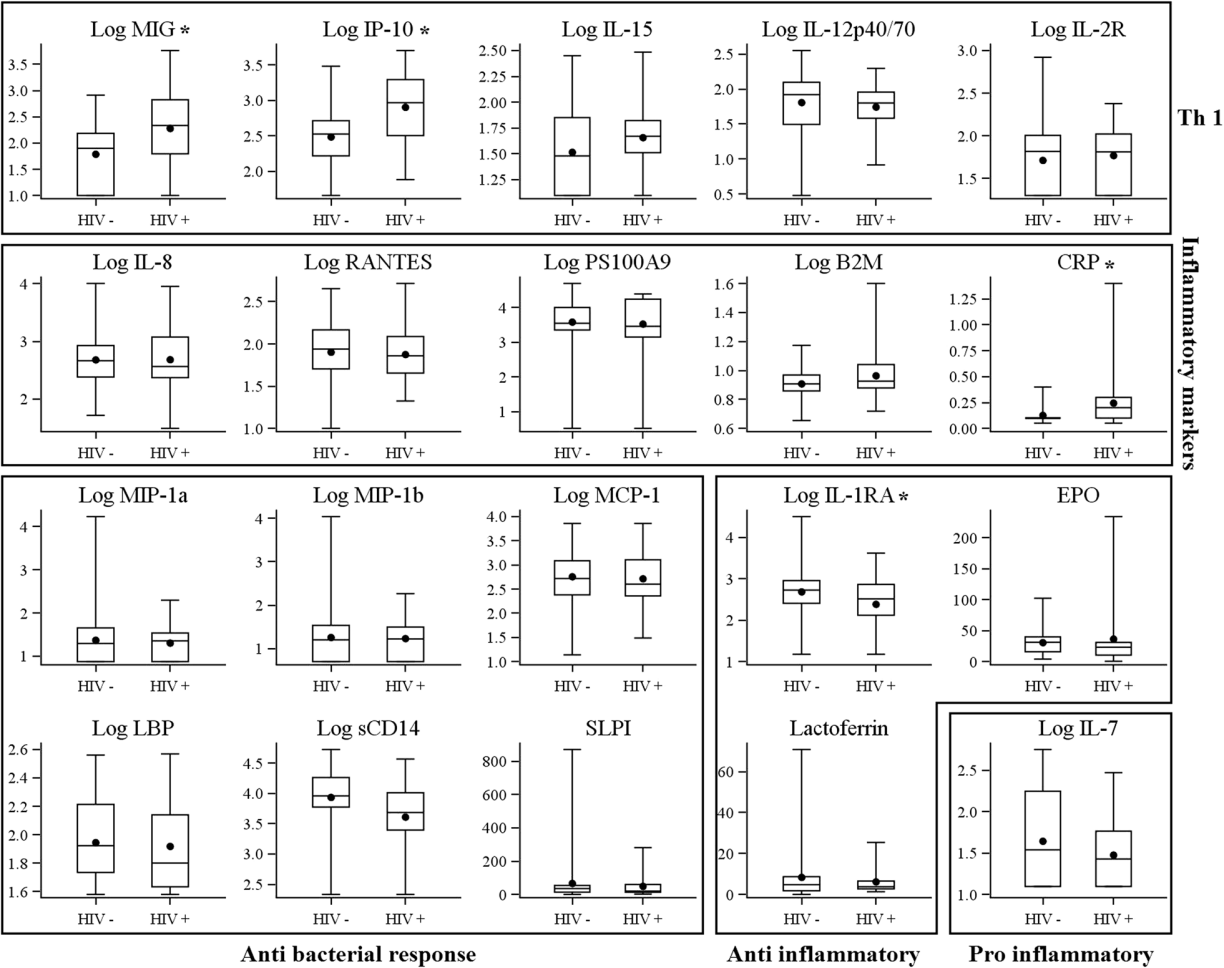
Breast milk immune factor comparisons between samples without sub-clinical mastitis from HIV-uninfected and HIV-infected women. Sub-clinical mastitis is defined as a Na/K ratio > 1 in breast milk. *Corrected p-value < 0.05. All concentrations are log-transformed for better readability except CRP, Lactoferrin, SLPI and EPO. All concentrations are in pg/mL except SLPI, B2M, CRP (μg/L), Lactoferrin (g/L), EPO (mIU/mL), LBP, and sCD14 (ng/mL)

The association between maternal HIV status and breast milk immune environment was explored by comparing concentrations of immune factors in samples without SCM (Fig. 1). The concentrations of immune factors were similar in the HIV-infected and uninfected women, except for MIG, IP-10 and CRP which were higher in HIV-infected women (respectively median [IQR]: MIG = 215 pg/mL [62–668] vs 79 pg/mL [10–153], corrected p = 0.002; median IP-10 = 932 pg/mL [320–1976] vs 337 pg/mL [165–519], corrected p = 0.001 and median CRP = 0.2 μg/L [0.1–0.3] vs 0.1 μg/L [0.1–0.1], corrected p = 0.001). In contrast, the level of IL-1RA was significantly lower in HIV-infected women (326 pg/mL [131–731] vs 531 pg/mL [255–905], corrected p = 0.045). A trend was observed towards higher B2M and lower sCD14 concentration in HIV-infected women (corrected p = 0.087, for each).

Multivariate mixed models, taking into account the clustering of left and right breast milk samples of a woman and adjusted on infant age at sampling, confirmed the association between HIV infection and MIG, IP-10, IL-1RA and CRP, as well as B2M and sCD14 (see Additional file 2: Table S2 for multivariate mixed models assessing the effect of HIV infection on breast milk immune factors, on samples without sub-clinical mastitis).

**Fig. 2:**
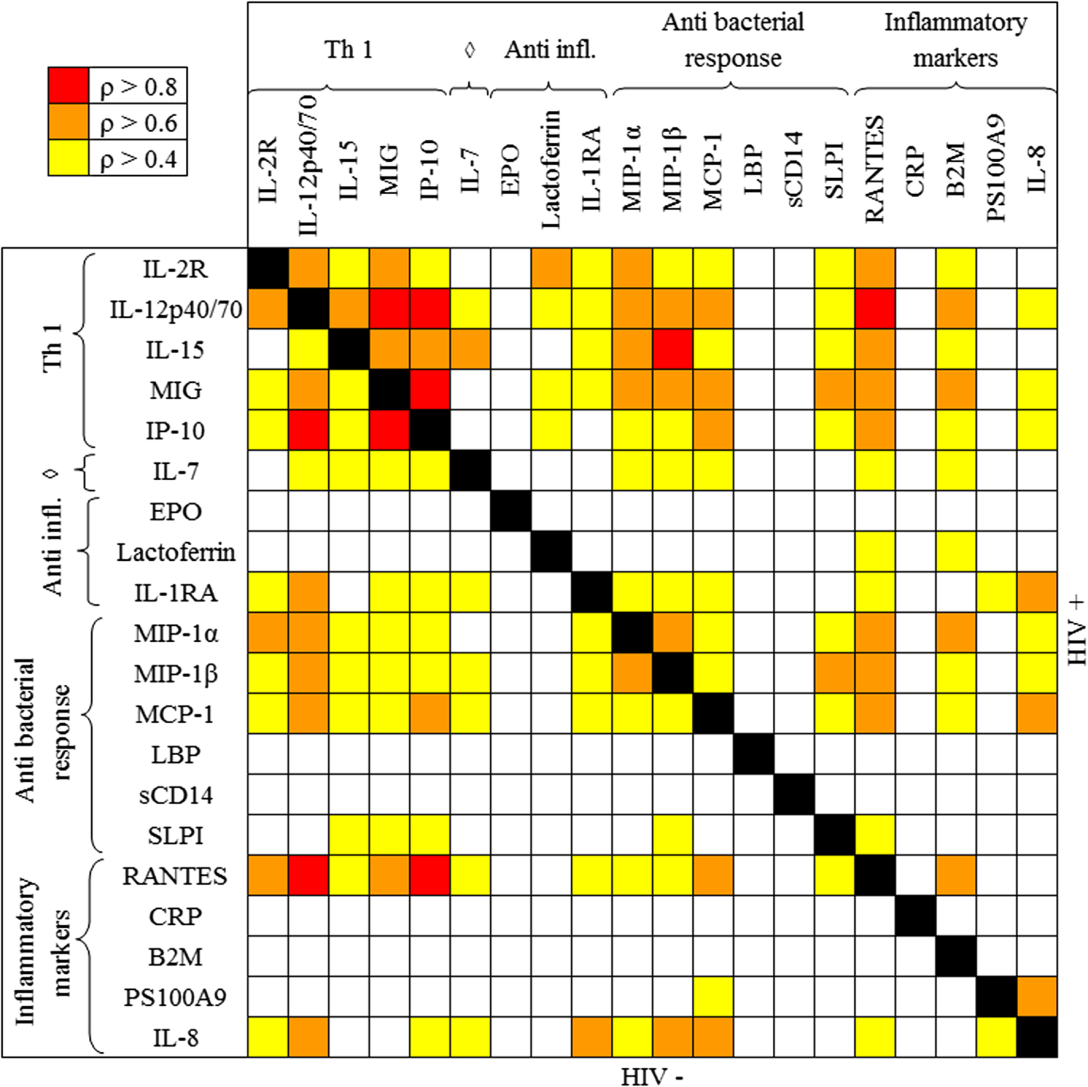
Spearman’s correlations between breast milk immune factor concentrations in samples without sub-clinical mastitis, by group. Sub-clinical mastitis is defined as a Na/K ratio > 1 in breast milk. ^◊^Pro inflammatory marker. Results for each HIV group are located either side of the black diagonal: below for the HIV-uninfected group and above for the HIV-infected group. Significant (p < 0.05) and noticeable (ρ > 0.4) correlations are represented by their intensity following color. All reported correlations were significant and positive

Correlations between the factors were analyzed to explore the immune network pattern in SCM-negative samples (Fig. 2). Significant and strong correlations were found between Th1-related cytokines in breast milk samples collected from HIV-infected women, especially between CXC chemokines secreted in response to IFN-γ: MIG and IP-10 (ρ = 0.86, p < 0.001), IP-10 and IL-12p40/70 (ρ = 0.84, p < 0.001) and between Th1-related cytokines and Th1-related CC chemokines, such as RANTES with both IL-12p40/70 (ρ = 0.82, p < 0.001) and IP-10 (ρ = 0.82, p < 0.001). The pattern of correlations was similar in samples of HIV-infected women with significant and strong correlations between IP-10 and both MIG (ρ = 0.87, p < 0.001) and IL-12p40/70 (ρ = 0.81, p < 0.001), MIG and IL-12p40/70 (ρ = 0.87, p < 0.001), between RANTES and IL-12p40/70 (ρ = 0.91, p < 0.001) and between IL-15 and MIP-1β (ρ = 0.85, p < 0.001).

We found only a few weak correlations (maximum |ρ| = 0.44) between breast milk immune factors and plasma HIV viral load, CD4 count or breast milk HIV RNA (see Additional file 3: Table S3 for correlations between breast milk immune factor concentration, HIV plasma parameters and breast milk HIV RNA).

### Association between HIV infection and breast milk immune factors in the presence of SCM

The concentrations of immune factors in samples with SCM were similar in the samples of HIV-infected and uninfected women (see Additional file 4: Table S4 for breast milk immune factor comparisons between samples with sub-clinical mastitis from HIV-uninfected and HIV-infected women).

In HIV-uninfected women, the concentrations of 14 out of 20 immune factors in breast milk were significantly higher in samples with SCM compared to samples without SCM. Differences remained significant after correcting p-values for multiple testing. Hence, Th1-related cytokines (IL-2 receptor, IL-12p40/70, IL-15, MIG and IP-10), factors secreted in response to bacterial exposure (MIP-1α, MIP-1β, MCP-1, SLPI) and inflammatory markers (RANTES, B2M, PS100A9 and IL-8) were significantly elevated in SCM-positive samples compared to SCM-negative samples. Among anti-inflammatory markers, only IL-1RA was increased. A trend was observed for higher LBP and sCD14 concentrations (Table 2).

**Table 2:** Comparisons of breast milk immune factor concentration with or without sub-clinical mastitis, by HIV group

Immune factor concentration	HIV+ samples		Corrected p-value*	HIV− samples		Corrected p-value*
	No SCM Median [q25–q75]	SCM Median [q25–q75]		No SCM Median [q25–q75]	SCM Median [q25–q75]	
Th 1						
IL-2R	65 [20–105]	82 [66–109]	0.251	66 [20–101]	166 [66–536]	0.008
IL-12p40/70	63 [38–91]	110 [57–126]	*0.030*	83 [31–126]	212 [100–333]	0.003
IL-15	47 [32–66]	77 [49–126]	0.102	30 [13–71]	100 [27–262]	0.021
MIG	215 [62–668]	442 [207–1077]	0.102	79 [10–153]	1185 [314–1328]	< *0.001*
IP-10	932 [320–1976]	1913 [557–2653]	0.102	337 [165–519]	1806 [502–3379]	< *0.001*
◊ IL-7	27 [13–58]	62 [26–109]	0.088	35 [13–177]	179 [13–279]	0.131
Anti infl.						
EPO	23 [10–31]	17 [13–33]	0.970	31 [16–40]	25 [18–33]	0.920
Lactoferrin	3.8 [2.8–6.6]	4.5 [2.8–13.8]	0.598	4.8 [2.2–8.7]	18.8 [2.8–20.2]	0.136
IL-1RA	326 [131–731]	430 [232–1203]	0.183	531 [255–905]	1718 [470–3980]	*0.012*
Anti bact. response						
MIP-1α	23 [8–34]	34 [24–137]	*0.030*	20 [8–45]	145 [20–808]	*0.007*
MIP-1β	17 [5–31]	32 [11–141]	0.102	16 [5–34]	163 [24–801]	*0.003*
MCP-1	400 [228–1281]	690 [338–1713]	0.224	524 [240–1221]	6460 [1994–7200]	*0.003*
LBP	63 [43–138]	248 [97–394]	*0.036*	84 [54–164]	357 [85–550]	0.064
sCD14	4828 [2479–10,231]	6573 [2560–9186]	0.845	9059 [5941–18,212]	27,778 [10,650–57,686]	0.064
SLPI	20 [13–60]	40 [32–82]	0.206	35 [14–54]	102 [55–562]	*0.011*
Infl. markers						
RANTES	72 [45–122]	151 [88–268]	*0.030*	87 [51–147]	238 [110–304]	*0.006*
CRP	0.20 [0.10–0.7]	0.20 [0.10–0.70]	0.591	0.10 [0.10–0.10]	0.10 [0.10–0.20]	0.203
B2M	8.5 [7.6–11.0]	10.1 [8.8–13.0]	0.088	8.1 [7.2–9.3]	13.6 [10.3–26.8]	< *0.001*
PS100A9	2875 [1405–17,183]	18,403 [4166–23,934]	0.088	3480 [2235–9930]	23,129 [8035–25,854]	*0.021*
IL-8	365 [233–1188]	1275 [503–2803]	*0.030*	460 [240–844]	3243 [414–10,160]	*0.007*

Sub-clinical mastitis is defined as a Na/K ratio > 1 in breast milk

[q25–q75] interquartile range

◊: pro inflammatory marker; Infl. markers: inflammatory markers; Anti infl.: anti inflammatory markers; anti bact. response: anti bacterial response

All concentrations are in pg/mL except SLPI, B2M, CRP (μg/L), lactoferrin (g/L), EPO (mIU/mL), LBP, sCD14 (ng/mL)

* p-values are for the test of the difference between samples with and without SCM, separately for each HIV group; Italic values indicate significance of p-value (<0.05) after FDR correction

The multivariate mixed models, taking into account the clustering of left and right breast milk samples of a woman and adjusted on infant age at sampling, confirmed the significant association between SCM and higher concentrations of breast milk IL-12p40/70, MIG, IP-10, IL-1RA, MCP-1, SLPI, B2M, and IL-8 in HIV-uninfected women (see Additional file 5: Table S5 for multivariate mixed models assessing the effect of SCM on breast milk immune factors, by HIV group).

In HIV-infected women, breast milk concentrations of five immune factors were significantly higher in SCM-positive samples than in SCM-negative samples: IL-12p40/70, MIP-1α, LBP, RANTES and IL-8 and a trend was observed for IL-7, B2M and PS100A9 (see Table 2). Interpretable multivariate mixed models confirmed the significant effect of SCM on higher concentrations of IL-12p40/70, MIP-1α, LBP, RANTES and IL-8, as well as IL-1RA and MIP-1β (see Additional file 5: Table S5).

## Discussion

We provided the first comparative analysis of a large number of soluble immune factors in the breast milk of ART-naive HIV-infected women and uninfected women and highlighted the association between HIV infection and immune response to SCM. Our study was performed on samples collected years ago, before the era of universal, life-long maternal ART, offering a unique opportunity to better understand the interactions between HIV infection and inflammation in the mammary gland without interference of therapy. Although pregnant and lactating women have today increasing access to ART, HIV replication during lactation still occurs in cases of virological failure or poor adherence, and in HIV-infected women unaware of their status. Furthermore, data collected before the era of ART are necessary to further explore the immune response to SCM in HIV-infected women undetectable for HIV on ART, in whom SCM may still occur, especially during weaning.

Our results showed that HIV infection moderately alters the soluble immune factor environment in breast milk samples without SCM or any other breast health problem. Hence, in the absence of SCM, the concentrations of immune factors in breast milk appeared different between HIV-uninfected women and HIV-infected women who didn’t transmit the infection by breastfeeding, regarding MIG and IP-10 that were significantly higher in the breast milk of HIV-infected women. These two CXC-chemokines are induced by IFN-γ and belong to a Th1 response and a cascade that is critical in antiviral defense. In addition, the level of the anti-inflammatory IL-1RA cytokine was significantly lower. Furthermore, the network of soluble immune factors appeared only slightly impaired, with correlations between the Th1 cytokines themselves and with the inflammatory markers that were slightly higher in the samples of HIV-positive women.

Three other studies who did not exclude women having transmitted HIV to their infants have assessed the impact of HIV infection on breast milk immune profile regardless of the presence of a SCM. Bosire et al. who compared MIP-1α, MIP-1β, RANTES and Stromal cell-Derived Factor-1α (SDF-1α) in the breast milk of Kenyan women several times after childbirth, found that MIP-1β was significantly higher at 10 days and RANTES at 1 month among HIV-infected versus HIV-uninfected women [39]. Shapiro et al. found significantly higher levels of total IgM, IgG, IgA and SLPI in HIV-infected women compared to HIV-uninfected women in Botswana [40]. Henrick et al. found significantly higher levels of soluble toll-like receptor 2 (sTLR2) in HIV-infected women [41].

SCM can be viewed as an initial stage of infection and inflammation that carries a risk of subsequent progression to a more severe mastitis [19]. Regarding the low frequency of mastitis, the immune response in the mammary gland is most of the time able to prevent the adverse evolution of SCM into symptomatic mastitis. Hence, only a few women reported clinical mastitis (1% in HIV-infected and 0.5% in HIV-uninfected women) or other breast health problems in the VTS cohort [24, 53]. In our study, approximately a quarter of HIV-uninfected women and a third of HIV-infected women had SCM, which is consistent with other studies conducted in both HIV-infected and uninfected women [16, 17, 19, 54].

Previous studies have explored breast milk immune factors during SCM (Na/K ratio > 1). Increased IL-8, lactoferrin, SLPI and RANTES were observed in HIV-uninfected women with SCM [16, 17, 54]. We recently explored breast milk environment during SCM in HIV-uninfected mothers from the VTS cohort. Our findings indicated that SCM is associated with higher levels of B2M, PS100A9, TNF-α, IL-6, IL-8, IL-17, RANTES, IL-2R, IL-12p40/70, IFN-α, IFN-γ, MIG and IP-10 [19]. All these results highlighted a robust, prompt and predominant Th1 and pro-inflammatory response to SCM in HIV-uninfected women. Our data confirmed that, in the presence of SCM, breast milk immune environment of HIV-uninfected women was characterized by a robust immune response involving a broad panel of Th1 and inflammatory related immune factors, as well as anti-bacterial response. By comparison, the breast milk immune factor environment appeared severely impaired during SCM in HIV-infected women who didn’t transmit HIV to their child by breastfeeding, with only five immune factors that were significantly increased compared to fourteen parameters in HIV-uninfected women. Furthermore, the magnitude of immune factors concentration differences in the presence of SCM wa slower in HIV-infected women compared to HIV-uninfected women.

These findings suggest that HIV infection is associated with a chronic stimulation of the Th1-related cytokines cascade in breast milk and an impaired ability to respond to SCM. The local inflammation of the mammary gland during SCM modulates both breast milk cell-free and cell-associated HIV levels and was found to increase HIV transmission through breastfeeding in several studies [25–30]. SCM is probably involved in mechanisms fuelling local viral replication and traffic of infected cells in the mammary gland from the vascular compartment [31, 32]. Cytomegalovirus and Epstein–Barr virus that are part of the normal environment of the mammary gland can also facilitate breast milk HIV shedding and were found associated with HIV-1 transmission by breastfeeding in the same cohort [33]. Furthermore, in a recent study we reported that impaired capacity to secret IL-8 in breast milk during SCM was associated with detection of Epstein–Barr virus in breast milk from HIV-infected Zambian women which may in turn fuel HIV shedding [34].

As a cross-sectional study the absence of follow-up is one of the limitations of our study. Infant age at sampling varied, but all specimens were mature breast milk and multivariate analysis took into account this possible confounding factor known to influence immunologic environment in the mammary gland. In addition, the relative homogeneity of our study population was a strength to address the issue of the multiple environmental factors that influence the immune composition of breast milk [55]. Exclusion of mother having transmitted HIV by breastfeeding may be viewed as a selection bias regarding the global population of HIV infected women. Based on the Vertical Transmission Study, the overall risk of postnatal HIV infection has been estimated at 3.9% among children breastfed for less than 6 months, each additional month of breastfeeding beyond 6 months of age being associated with a 1% risk of acquisition of HIV [56]. To be representative of the global population of HIV-infected mothers at risk of postnatal transmission, our study should have include an estimated number of two to three mothers having transmitted HIV infection by breastfeeding. However, we did not include women who transmitted HIV to their infant postnatally, assuming that the soluble immunologic pattern in breast milk from HIV-transmitters could form a peculiar type of pattern, as mentioned by several authors [46, 57–59].

## Conclusion

HIV infection is associated with a raise of breast milk IP-10 and MIG concentrations, which are cytokines induced by IFN-γ and belong to antiviral defense. Results of this study including only women who didn’t transmit HIV by breastfeeding suggest that during SCM, the breast milk environment is characterized by a lower and narrower immune response associated with maternal HIV infection. Further studies including women having transmitted HIV in the postnatal period are needed to confirm that the capacity of the mammary gland to face SCM is impaired in HIV-infected women and may contribute to facilitate HIV transmission by breastfeeding.

## Additional files

**Additional file 1: Table S1.** Detection rates of immune factors in samples with and without sub-clinical mastitis, by HIV group. This table compares the detection rates of immune factors measured in mature breast milk samples with and without sub-clinical mastitis, separately for samples from HIV-infected and HIV-uninfected women. There were no detection rate differences between samples with and without SCM in samples from HIV-infected women. In comparison, in samples from HIV-uninfected women, interferon-α, interferon-γ, interleukin-4, α-defensin, Tumor Necrosis Factor-α and interleukin-6 were more frequently detected in samples with SCM compared to samples without SCM.

**Additional file 2: Table S2.** Multivariate models assessing the effect of HIV on immunologic factor concentration in samples without SCM. This table indicates the adjusted regression coefficients and associated p-values of multivariate mixed linear models assessing the effect of HIV infection on each breast milk soluble immunologic factor concentration, in samples without sub-clinical mastitis, adjusted on child age at the time of sampling. HIV infection was associated with an increase of monokine induced by gamma interferon, inflammatory protein-10, C-reactive protein and ß2-microglobuline, and a decrease of receptor antagonist of interleukin 1ß and soluble CD14.

**Additional file 3: Table S3.** Correlations between breast milk immune factor concentration, HIV plasma parameters and breast milk HIV RNA. This table indicates the Spearman’s non parametric correlations and associated p-values between breast milk soluble immunologic factor concentration and plasma HIV viral load (approximately 6 months after delivery), plasma CD4 count (approximately 6 months after delivery) and breast milk HIV RNA (at the time of breast milk sampling), in samples from HIV-infected women only. There are only a few weak correlations (maximum |ρ| = 0.44).

**Additional file 4: Table S4.** Breast milk immune factor comparisons between samples with sub-clinical mastitis from HIV-uninfected and HIV-infected women. This table compares the concentration of immune factors measured in mature breast milk samples with sub-clinical mastitis, between samples from HIV-infected and HIV-uninfected women. There were no statistically significant concentration differences in samples with sub-clinical mastitis between HIV-infected and HIV-uninfected women.

**Additional file 5: Table S5.** Multivariate models assessing the effect of SCM on immunologic factor concentration, by HIV group. This table indicates the adjusted regression coefficients and associated p-values of multivariate mixed linear models assessing the effect of sub-clinical mastitis on each breast milk soluble factor concentration, adjusted on child age at the time of sampling, separately for samples from HIV-infected and HIV-uninfected women. Sub-clinical mastitis was associated with and increase of 9/13 immune factors analyzed in samples from HIV-uninfected women compared to 7/17 immune factors analyzed in samples from HIV-infected women.

## Abbreviations

ANRS: Agence Nationale de Recherche sur le SIDA
ART: antiretroviral therapy
B2M: β2microglobuline
CD4: cluster of differentiation 4
CRP: C-reactive protein
EBF: exclusive breastfeeding
EPO: erythropoietin
FDR: false discovery rate
GM-CSF: granulocyte and macrophage growth factor
HIV: human immunodeficiency virus
IFN: interferon
Ig: immunoglobulin
IL: interleukin
IL-1RA: receptor antagonist of interleukin 1β
IL-2R: interleukin 2 receptor
IP: inflammatory protein
LBP: lipopolysaccharide-binding protein
MBF: mixed breastfeeding
MCP: monocyte chemotactic protein
MIG: monokine induced by gamma interferon
MIP: macrophage inflammatory protein
MTCT: mother-to-child HIV transmission
PS100A9: S100A9 protein
RANTES: regulated upon activation normal T-cell expressed and secreted
RNA: ribonucleic acid
sCD14: soluble CD14
SCM: subclinical mastitis
SDF-1α: stromal cell-derived factor-1α
SLPI: secretory leukocyte peptidase inhibitor
sTLR2: soluble toll-like receptor 2
TH: T helper
TNF-α: tumor necrosis factor-α
VTS: vertical transmission study
WHO: World Health Organization
