# Supplementary material for: The immune response to sub-clinical mastitis is impaired in HIV-infected women
Source: J Transl Med. 2018 Oct 25;16:296. doi: 10.1186/s12967-018-1667-4 (PMC6202806; doi:10.1186/s12967-018-1667-4)
Supplement: Supplementary file 1 — Additional file 1: Table S1. Detection rates of immune factors in samples with and without sub-clinical mastitis, by HIV group. This table compares the detection rates of immune factors measured in mature breast milk samples with and without sub-clinical mastitis, separately for samples from HIV-infected and HIV-uninfected women. There were no detection rate differences between samples with and without SCM in samples from HIV-infected women. In comparison, in samples from HIV-uninfected women, interferon-α, interferon-γ, interleukin-4, α-defensin, Tumor Necrosis Factor-α and interleukin-6 were more frequently detected in samples with SCM compared to samples without SCM. [file 12967_2018_1667_MOESM1_ESM.docx]

**Additional Table S1. Detection rates of immune factors in samples with and without sub-clinical mastitis, by HIV group**

| **Immune factor detection rate** | **HIV + samples** | |  | **HIV – samples** | |  |
| --- | --- | --- | --- | --- | --- | --- |
|  | **No SCM**  Nb/total (%) | **SCM**  Nb/total (%) | Corrected p-value* | **No SCM**  Nb/total (%) | **SCM**  Nb/total (%) | Corrected p-value* |
| IL-2 | 1/54 (1.9) | 0/18 (0) | 1 | 0/67 (0) | 1/14 (7.1) | 0.300 |
| IL-2 receptor | 39/54 (72.2) | 15/18 (83.3) | 0.616 | 36/67 (53.7) | 11/13 (84.6) | 0.111 |
| IL-12p40/70 | 54/54 (100) | 18/18 (100) | . | 65/67 (97.0) | 14/14 (100.0) | 1 |
| IL-15 | 45/54 (83.3) | 17/18 (94.4) | 0.616 | 35/67 (52.2) | 10/13 (76.9) | 0.238 |
| IFN-α | 1/54 (1.9) | 1/18 (5.6) | 0.616 | **3/67 (4.5)** | **6/13 (46.2)** | **0.003** |
| IFN-γ | 5/55 (9.1) | 1/18 (5.6) | 1 | **0/67 (0)** | **3/14 (21.4)** | **0.018** |
| MIG | 47/54 (87.0) | 18/18 (100) | 0.283 | 49/67 (73.1) | 13/13 (100) | 0.111 |
| IP-10 | 54/54 (100) | 18/18 (100) | . | 67/67 (100) | 14/14 (100) | . |
| IL-4 | 7/54 (13.0) | 6/18 (33.3) | 0.227 | **1/67 (1.5)** | **5/14 (35.7)** | **0.003** |
| IL-5 | 0/54 (0) | 1/18 (5.6) | 0.525 | 0/67 (0) | 0/13 (0) | . |
| IL-13 | 0/54 (0) | 2/18 (11.1) | 0.227 | 0/67 (0) | 1/14 (7.1) | 0.300 |
| IL-17 | 0/54 (0) | 0/18 (0) | . | 0/67 (0) | 113 (7.7) | 0.300 |
| IL-7 | 28/54 (51.9) | 14/18 (77.8) | 0.227 | 34/67 (50.8) | 8/13 (61.5) | 0.728 |
| GM-CSF | 0/54 (0) | 0/18 (0) | . | 3/67 (4.5) | 1/13 (7.7) | 0.728 |
| IL-10 | 1/54 (1.9) | 1/18 (5.6) | 0.616 | 2/67 (3.0) | 3/14 (21.4) | 0.111 |
| EPO | 1/17 (100) | 5/5 (100) | . | 24/24 (100) | 6/6 (100) | . |
| Lactoferrin | 27/27 (100) | 8/8 (100) | . | 33/34 (97.1) | 9/9 (100) | 1 |
| IL-1RA | 47/54 (87.0) | 16/18 (88.9) | 1 | 66/67 (98.5) | 13/13 (100) | 1 |
| MIP-1α | 36/54 (66.7) | 16/18 (88.9) | 0.227 | 41/67 (61.2) | 11/14 (78.6) | 0.353 |
| MIP-1β | 40/54 (74.1) | 15/18 (83.3) | 0.700 | 45/67 (67.2) | 11/14 (78.6) | 0.728 |
| MCP-1 | 54/54 (100) | 18/18 (100) | . | 67/67 (100) | 13/13 (100) | . |
| LBP | 22/22 (100) | 9/9 (100) | . | 31/31 (100) | 6/6 (100) | . |
| sCD14 | 23/23 (100) | 10/10 (100) | . | 33/33 (100) | 9/9 (100) | . |
| SLPI | 25/25 (100) | 8/8 (100) | . | 31/32 (96.9) | 9/9 (100) | 1 |
| α-defensin | 7/25 (28.0) | 4/8 (50.0) | 0.616 | **11/35 (31.4)** | **9/10 (90.0)** | **0.012** |
| RANTES | 55/55 (100) | 19/19 (100) | . | 63/67 (94.0) | 13/14 (92.9) | 1 |
| EOTAXIN | 5/54 (9.3) | 7/18 (38.9) | 0.130 | 4/67 (6.0) | 3/13 (23.1) | 0.210 |
| CRP | 54/55 (98.2) | 19/19 (100) | 1 | 62/67 (92.5) | 13/15 (86.7) | 0.788 |
| B2M | 55/55 (100) | 19/19 (100) | . | 67/67 (100) | 15/15 (100) | . |
| PS100A9 | 23/24 (95.8) | 9/9 (100) | 1 | 30/31 (96.8) | 8/8 (100) | 1 |
| TNF-α | 1/54 (1.9) | 4/18 (22.2) | 0.130 | **2/67 (3.0)** | **7/13 (53.9)** | **0.0004** |
| IL-6 | 20/54 (37.0) | 12/18 (66.7) | 0.199 | **12/67 (17.9)** | **10/13 (76.9)** | **0.0009** |
| IL-8 | 54/54 (100) | 18/18 (100) | . | 67/67 (100) | 14/14 (100) | . |
| IL-1β | 24/54 (44.4) | 11/18 (61.1) | 0.514 | 16/67 (23.9) | 6/13 (46.2) | 0.300 |

Sub-clinical mastitis is defined as a Na/K ratio>1 in breast milk.

*P-values are for the test of the difference between samples with and without SCM, separately for each HIV group; p-values are corrected for False Discovery Rate.

This table compares the detection rates of immune factors measured in mature breast milk samples with and without sub-clinical mastitis, separately for samples from HIV-infected and HIV-uninfected women. There were no detection rate differences between samples with and without SCM in samples from HIV-infected women. In comparison, in samples from HIV-uninfected women, interferon-α, interferon-γ, interleukin-4, α-defensin, Tumor Necrosis Factor-α and interleukin-6 were more frequently detected in samples with SCM compared to samples without SCM.
