# Supplementary material for: The immune response to sub-clinical mastitis is impaired in HIV-infected women
Source: J Transl Med. 2018 Oct 25;16:296. doi: 10.1186/s12967-018-1667-4 (PMC6202806; doi:10.1186/s12967-018-1667-4)
Supplement: Supplementary file 2 — Additional file 2: Table S2. Multivariate models assessing the effect of HIV on immunologic factor concentration in samples without SCM. This table indicates the adjusted regression coefficients and associated p-values of multivariate mixed linear models assessing the effect of HIV infection on each breast milk soluble immunologic factor concentration, in samples without sub-clinical mastitis, adjusted on child age at the time of sampling. HIV infection was associated with an increase of monokine induced by gamma interferon, inflammatory protein-10, C-reactive protein and ß2-microglobuline, and a decrease of receptor antagonist of interleukin 1ß and soluble CD14. [file 12967_2018_1667_MOESM2_ESM.docx]

**Additional Table S2. Multivariate models assessing the effect of HIV on immunologic factor concentration in samples without SCM**

| **Immune factor** | **Adjusted regression coefficient [95% CI]** | **P-value** |
| --- | --- | --- |
| IL-12p40/70 | -0.07 [-0.22;0.08] | 0.377 |
| MIG | 0.48 [0.20;0.75] | **<0.001** |
| IP-10 | 0.42 [0.24;0.60] | **<0.001** |
| EPO ♦ | -0.17 [-0.50;0.17] | 0.321 |
| IL-1RA | -0.33 [-0.58;-0.08] | **0.010** |
| MCP-1 | -0.04 [-0.26;0.17] | 0.700 |
| LBP ♦ | -0.02 [-0.19;0.15] | 0.790 |
| sCD14 ♦ | -0.32 [-0.62;-0.02] | **0.039** |
| SLPI ♦ | -0.02 [-0.34;0.31] | 0.922 |
| RANTES | -0.03 [-0.17;0.10] | 0.618 |
| CRP | 0.24 [0.13;0.35] | **<0.001** |
| B2M | 0.06 [0.01;0.10] | **0.020** |
| IL-8 | 0.004 [-0.18;0.18] | 0.962 |

Sub-clinical mastitis is defined as a Na/K ratio>1 in breast milk.

All concentrations are log transformed.

All concentrations are in pg/mL except SLPI, B2M, EPO (mIU/mL) and sCD14 (ng/mL).

♦Model without random effect because of low number of paired samples.

Uninterpretable models are not presented because of heteroscedasticity and residuals not normally distributed.

This table indicates the adjusted regression coefficients and associated p-values of multivariate mixed linear models assessing the effect of HIV infection on each breast milk soluble immunologic factor concentration, in samples without sub-clinical mastitis, adjusted on child age at the time of sampling. HIV infection was associated with an increase of Monokine Induced by Gamma interferon, Inflammatory Protein-10, C-Reactive Protein and ß2-microglobuline, and a decrease of Receptor Antagonist of Interleukin 1ß and soluble CD14.
