# Supplementary material for: The immune response to sub-clinical mastitis is impaired in HIV-infected women
Source: J Transl Med. 2018 Oct 25;16:296. doi: 10.1186/s12967-018-1667-4 (PMC6202806; doi:10.1186/s12967-018-1667-4)
Supplement: Supplementary file 3 — Additional file 3: Table S3. Correlations between breast milk immune factor concentration, HIV plasma parameters and breast milk HIV RNA. This table indicates the Spearman’s non parametric correlations and associated p-values between breast milk soluble immunologic factor concentration and plasma HIV viral load (approximately 6 months after delivery), plasma CD4 count (approximately 6 months after delivery) and breast milk HIV RNA (at the time of breast milk sampling), in samples from HIV-infected women only. There are only a few weak correlations (maximum |ρ| = 0.44). [file 12967_2018_1667_MOESM3_ESM.docx]

**Additional Table S3. Correlations between breast milk immune factor concentration, HIV plasma parameters and breast milk HIV RNA**

|  | **Plasma HIV viral load** | | | **CD4 count** | | | **Breast milk HIV RNA** | | |
| --- | --- | --- | --- | --- | --- | --- | --- | --- | --- |
|  | Spearman's ρ | p-value | n | Spearman's ρ | p-value | n | Spearman's ρ | p-value | n |
| **IL-2R** | 0.10 | 0.28 | 111 | -0.07 | 0.45 | 115 | 0.17 | 0.23 | 52 |
| **IL-12p40/70** | -0.02 | 0.84 | 111 | 0.04 | 0.69 | 115 | **0.29** | **0.03** | 52 |
| **IL-15** | **0.19** | **0.04** | 111 | **-0.31** | **<.001** | 115 | 0.28 | 0.05 | 52 |
| **MIG** | **0.36** | **<.0001** | 111 | **-0.27** | **<.001** | 115 | 0.26 | 0.06 | 52 |
| **IP-10** | **0.44** | **<.0001** | 111 | **-0.33** | **<.001** | 115 | 0.27 | 0.05 | 52 |
| **IL-7** | -0.01 | 0.90 | 111 | 0.04 | 0.70 | 115 | 0.18 | 0.20 | 52 |
| **EPO** | -0.05 | 0.79 | 38 | 0.11 | 0.48 | 40 | . | . | 16 |
| **Lactoferrin** | 0.01 | 0.96 | 55 | -0.08 | 0.58 | 58 | 0.13 | 0.53 | 25 |
| **MIP-1a** | 0.02 | 0.86 | 111 | -0.03 | 0.78 | 115 | 0.13 | 0.37 | 52 |
| **MIP-1b** | 0.02 | 0.80 | 111 | -0.15 | 0.11 | 115 | 0.23 | 0.10 | 52 |
| **MCP-1** | 0.04 | 0.65 | 111 | 0.04 | 0.63 | 115 | 0.15 | 0.28 | 52 |
| **LBP** | -0.16 | 0.27 | 48 | -0.13 | 0.37 | 50 | . | . | 22 |
| **sCD14** | -0.18 | 0.20 | 51 | 0.16 | 0.26 | 53 | 0.26 | 0.24 | 23 |
| **SLPI** | -0.14 | 0.31 | 52 | 0.03 | 0.81 | 54 | 0.31 | 0.15 | 23 |
| **RANTES** | -0.01 | 0.89 | 112 | -0.03 | 0.77 | 116 | **0.33** | **0.02** | 53 |
| **CRP** | **0.27** | **<.001** | 112 | **-0.31** | **<.001** | 116 | 0.07 | 0.63 | 53 |
| **B2M** | **0.24** | **0.01** | 112 | **-0.38** | **<.0001** | 116 | **0.28** | **0.04** | 53 |
| **ps100** | -0.13 | 0.38 | 50 | 0.23 | 0.10 | 52 | -0.25 | 0.24 | 24 |
| **IL-1RA** | -0.18 | 0.05 | 111 | 0.16 | 0.09 | 115 | -0.08 | 0.56 | 52 |
| **IL-8** | 0.01 | 0.98 | 111 | 0.12 | 0.22 | 115 | 0.12 | 0.40 | 52 |

This table indicates the Spearman’s non parametric correlations and associated p-values between breast milk soluble immunologic factor concentration and plasma HIV viral load (approximately 6 months after delivery), plasma CD4 count (approximately 6 months after delivery) and breast milk HIV RNA (at the time of breast milk sampling), in samples from HIV-infected women only. There are only a few weak correlations (maximum |ρ|=0,44).
