# Supplementary material for: The immune response to sub-clinical mastitis is impaired in HIV-infected women
Source: J Transl Med. 2018 Oct 25;16:296. doi: 10.1186/s12967-018-1667-4 (PMC6202806; doi:10.1186/s12967-018-1667-4)
Supplement: Supplementary file 4 — Additional file 4: Table S4. Breast milk immune factor comparisons between samples with sub-clinical mastitis from HIV-uninfected and HIV-infected women. This table compares the concentration of immune factors measured in mature breast milk samples with sub-clinical mastitis, between samples from HIV-infected and HIV-uninfected women. There were no statistically significant concentration differences in samples with sub-clinical mastitis between HIV-infected and HIV-uninfected women. [file 12967_2018_1667_MOESM4_ESM.docx]

**Additional Table S4. Breast milk immune factor comparisons between samples with sub-clinical mastitis from HIV-uninfected and HIV-infected women**

|  | | **SCM + samples** | | Corrected p-value* |
| --- | --- | --- | --- | --- |
|  |  | **HIV -** | **HIV +** |  |
|  |  | Median [q25-q75] | |  |
| Th 1 | IL-2R | 166 [66-536] | 82 [66-109] | 0.311 |
|  | IL-12p40/70 | 212 [100-333] | 110 [57-126] | 0.155 |
|  | IL-15 | 100 [27-262] | 77 [49-126] | 0.612 |
|  | MIG | 1185 [314-1328] | 442 [207-1077] | 0.521 |
|  | IP-10 | 1806 [502-3379] | 1913 [557-2653] | 0.822 |
| ◊ | IL-7 | 179 [13-279] | 62 [26-109] | 0.413 |
| Anti infl. | EPO | 25 [18-33] | 17 [13-33] | 0.581 |
|  | Lactoferrin | 18.8 [2.8-20.2] | 4.5 [2.8-13.8] | 0.575 |
|  | IL-1RA | 1718 [470-3980] | 430 [232-1203] | 0.212 |
| Anti bact. response | MIP-1α | 145 [20-808] | 34 [24-137] | 0.504 |
|  | MIP-1β | 163 [24-801] | 32 [11-141] | 0.442 |
|  | MCP-1 | 6460 [1994-7200] | 690 [338-1713] | 0.120 |
|  | LBP | 357 [85-550] | 248 [97-394] | 0.581 |
|  | sCD14 | 27778 [10650-57686] | 6573 [2560-9186] | 0.155 |
|  | SLPI | 102 [55-562] | 40 [32-82] | 0.312 |
| Infl. markers | RANTES | 238 [110-304] | 151 [88-268] | 0.521 |
|  | CRP | 0.10 [0.10-0.20] | 0.20 [0.10-0.70] | 0.240 |
|  | B2M | 13.6 [10.3-26.8] | 10.1 [8.8-13.0] | 0.155 |
|  | PS100A9 | 23129 [8035-25854] | 18403 [4166-23934] | 0.661 |
|  | IL-8 | 3243 [414-10160] | 1275 [503-2803] | 0.521 |

Sub-clinical mastitis is defined as a Na/K ratio>1 in breast milk.

◊Pro inflammatory marker; Infl. Markers: inflammatory markers; Anti infl.: anti inflammatory markers; Anti bact. response: anti bacterial response

[q25-q75] interquartile range

*P-values are for the test of the difference between samples with and without SCM, separately for each HIV group; p-values are corrected for False Discovery Rate.

All concentrations are in pg/mL except SLPI, B2M, CRP (μg/L), lactoferrin (g/L), EPO (mIU/mL), LBP, sCD14 (ng/mL).

This table compares the concentration of immune factors measured in mature breast milk samples with sub-clinical mastitis, between samples from HIV-infected and HIV-uninfected women. There were no statistically significant concentration differences in samples with sub-clinical mastitis between HIV-infected and HIV-uninfected women.
