# Supplementary material for: The immune response to sub-clinical mastitis is impaired in HIV-infected women
Source: J Transl Med. 2018 Oct 25;16:296. doi: 10.1186/s12967-018-1667-4 (PMC6202806; doi:10.1186/s12967-018-1667-4)
Supplement: Supplementary file 5 — Additional file 5: Table S5. Multivariate models assessing the effect of SCM on immunologic factor concentration, by HIV group. This table indicates the adjusted regression coefficients and associated p-values of multivariate mixed linear models assessing the effect of sub-clinical mastitis on each breast milk soluble factor concentration, adjusted on child age at the time of sampling, separately for samples from HIV-infected and HIV-uninfected women. Sub-clinical mastitis was associated with and increase of 9/13 immune factors analyzed in samples from HIV-uninfected women compared to 7/17 immune factors analyzed in samples from HIV-infected women. [file 12967_2018_1667_MOESM5_ESM.docx]

**Additional Table S5. Multivariate models assessing the effect of SCM on immunologic factor concentration, by HIV group**

| **Immune factor** | **HIV + samples** | | **HIV - samples** | |
| --- | --- | --- | --- | --- |
|  | **Adjusted regression coefficient [95% CI]** | **P-value** | **Adjusted regression coefficient [95% CI]** | **P-value** |
| IL-12p40/70 | 0.20 [0.02;0.39] | **0.034** | 0.39 [0.03;0.75] | **0.040** |
| IL-15 | 0.21 [-0.03;0.44] | 0.072 | . | **.** |
| MIG | 0.32 [-0.05;0.70] | 0.078 | 1.03 [0.55;1.52] | **0.004** |
| IP-10 | 0.21 [-0.02;0.44] | 0.062 | 0.63 [0.31;0.95] | **0.006** |
| IL-7 | 0.24 [-0.07;0.54] | 0.100 | 0.34 [-0.17;0.86] | 0.140 |
| EPO ♦ | . | . | 0.15 [-0.17;0.47] | 0.342 |
| Lactoferrin ♦ | 0.07 [-0.24;0.37] | 0.665 | . | . |
| IL-1 RA | 0.51 [0.05;0.97] | **0.036** | 0.53 [0.09;0.96] | **0.028** |
| MIP-1α | 0.50 [0.19;0.81] | **0.009** | . | **.** |
| MIP-1β | 0.57 [0.16;0.98] | **0.017** | . | **.** |
| MCP-1 | 0.23 [-0.15;0.61] | 0.182 | 0.67 [0.21;1.13] | **0.015** |
| LBP ♦ | 0.37 [0.10;0.65] | **0.010** | 0.41 [0.11;0.71] | **0.009** |
| sCD14 ♦ | 0.01 [-0.47;0.49] | 0.974 | 0.37 [-0.02;0.76] | 0.060 |
| SLPI ♦ | 0.27 [-0.11;0.65] | 0.161 | 0.66 [0.16;1.16] | **0.012** |
| RANTES | 0.27 [0.09;0.45] | **0.010** | 0.31 [-0.01;0.63] | 0.056 |
| B2M | 0.06 [-0.02;0.14] | 0.110 | 0.27 [0.15;0.39] | **0.003** |
| PS100A9 ♦ | 0.50 [-0.12;1.12] | 0.112 | . | . |
| IL-8 | 0.41 [0.04;0.77] | **0.035** | 0.68 [0.26;1.09] | **0.011** |

Sub-clinical mastitis is defined as a Na/K ratio>1 in breast milk.

All concentrations are log transformed.

All concentrations are in pg/mL except SLPI, B2M, lactoferrin (g/L), EPO (mIU/mL) and sCD14 (ng/mL).

♦Model without random effect because of low number of paired samples.

Uninterpretable models are not presented because of heteroscedasticity and residuals not normally distributed.

This table indicates the adjusted regression coefficients and associated p-values of multivariate mixed linear models assessing the effect of sub-clinical mastitis on each breast milk soluble factor concentration, adjusted on child age at the time of sampling, separately for samples from HIV-infected and HIV-uninfected women. Sub-clinical mastitis was associated with and increase of 9/13 immune factors analyzed in samples from HIV-uninfected women compared to 7/17 immune factors analyzed in samples from HIV-infected women.
